# Association between subfoveal choroidal thickness and leakage site on fluorescein angiography in Behçet’s uveitis

**DOI:** 10.1038/s41598-019-45149-4

**Published:** 2019-06-13

**Authors:** Shintaro Shirahama, Toshikatsu Kaburaki, Hisae Nakahara, Rie Tanaka, Keiko Komae, Yujiro Fujino, Hidetoshi Kawashima, Makoto Aihara

**Affiliations:** 1 Department of Ophthalmology, University of Tokyo Graduate School of Medicine, 7-3-1 Hongo, Bunkyo-ku, Tokyo 113-8655 Japan; 2 Department of Ophthalmology, Tokyo Shinjuku Medical Center, 5-1 Tsukudo-cho, Shinjuku-ku, Tokyo 162-8543 Japan; 3 Department of Ophthalmology, Jichi Medical University, 3311-1 Yakushiji, Shimotsuke-shi, Tochigi 329-0498 Japan

**Keywords:** Uveal diseases, Eye diseases

## Abstract

This study aimed to clarify the association between the retinal leakage site on fluorescein angiography (FA) and subfoveal choroidal thickness (SCT) measured using enhanced depth imaging optical coherence tomography (EDI-OCT). Twenty-two patients with Behçet’s uveitis were retrospectively selected in this study. They underwent EDI-OCT and FA in both the active and convalescent phases. The associations of the changes between the active and convalescent phases in SCT and in FA leakage in various retinal areas (total retina, peripheral retina, macula, and optic disc) were examined. The changing rates of SCT between the two investigated phases were significantly associated with the changes in total FA leakage scores (y = 1.79X+ 11.7, r^2^ = 0.210, p < 0.05). Furthermore, the changes in FA leakage scores in the macula were correlated with the changing rates in SCT (y = 3.72X+ 13.9, r^2^ = 0.219, p < 0.05). By contrast, there were no significant associations between the changes in SCT and those in leakage from the peripheral retina or the optic disc on FA. These findings demonstrate that SCT may reflect macular vasculitis as determined using FA, and SCT measurement could be a non-invasive method to investigate inflammation near the macula in Behçet’s uveitis.

## Introduction

Behçet’s disease (BD) is a systemic inflammatory disease characterised by oral and genital mucous ulcers, uveitis, and dermic lesions^1^. The ocular involvement is characterised by recurrent sudden attacks of intraocular inflammation, and has been reported to affect between 47.4% and 69.0% of BD patients in Japan^2^. The disease activity with ocular involvement is usually evaluated by the frequency of ocular attacks, the location of the inflammation site, and scales, such as the BD ocular attack score 24, which determine ocular inflammatory signs^3^.

Fluorescein angiography (FA) is a valuable tool for the assessment of inflammatory fundus conditions due to posterior uveitis^4^. A previous study demonstrated that both the frequency of ocular attacks and the background leakage from the peripheral retina, macula, and optic disc were significantly decreased with infliximab therapy^5^. Therefore, the leakage on FA may serve as an important surrogate marker for the inflammatory activity in BD uveitis^5^. However, FA is an invasive examination using an intravenous infusion of the dye fluorescein, which may be associated with serious complications such as an anaphylactic shock^6^. Thus, frequently in clinical practice, FA cannot be performed to evaluate the disease activity in ocular BD patients.

In contrast, optical coherence tomography (OCT) is a non-invasive tool used to visualise the posterior areas of the ocular fundus and is frequently used for the assessment of retinal diseases. However, conventional spectral-domain OCT devices have limitations in imaging the choroid because of decreased sensitivity and resolution due to several reasons such as wavelength-dependent light scattering and signal loss in the image path^7,8^. Enhanced depth imaging OCT (EDI-OCT) has made it possible to easily obtain detailed images of the choroid^8^. This technique has been used for the evaluation of disease activity and treatment effectiveness in choroidal inflammatory diseases such as the Vogt-Koyanagi-Harada disease^9^. Moreover, a recent retrospective study demonstrated that subfoveal choroidal thickness (SCT) measured with EDI-OCT is increased in the active phase compared to that in the convalescent phase in BD posterior uveitis^10^. In particular, the SCT reduction in the convalescent compared to the active phase was significantly correlated with an improvement in retinal vascular leakage revealed using FA^10^. This result suggests that SCT is correlated with retinal vascular leakage identified using FA. Thus, SCT measurements may be potentially useful for monitoring the activity in Behçet’s uveitis, rendering FA assessments redundant^10^.

To date, it remains unclear whether retinal FA leakage sites are correlated with SCT. In this study, we investigated the relationship between the leakage site in FA and SCT to examine whether SCT changes reflect FA extravasation in BD uveitis.

## Methods

In this retrospective study, the clinical records of 80 consecutive ocular BD patients who arrived at the Uveitis Clinic of the University of Tokyo Hospital from January 2013 to March 2019 were initially reviewed. BD was diagnosed based on the criteria established by the BD Research Committee of Japan^11^. The inclusion criteria of this study were as follows: (1) BD uveitis with evidence of inflammation involving the posterior segment (posterior uveitis) based on the Standardization of Uveitis Nomenclature (SUN) working group classification^12^, (2) treatment with colchicine, cyclosporine, or tumour necrosis factor-α inhibitors to prevent the recurrence of posterior uveitis, and (3) EDI-OCT and FA imaging performed in both the active and the convalescent phase. The active phase was defined as an acute exacerbation of posterior uveitis within 3 months according to the SUN working group definition^12^: an increase of at least two steps in the vitreous haze score with or without anterior chamber cells, new retinal exudates with or without haemorrhages, or a decrease of more than three lines in the Snellen visual acuity measurement. In contrast, the convalescent phase was defined as an inactive disease persisting for more than 3 months after resolution of an acute inflammation and without signs of a uveitis exacerbation including the development of retinitis, vasculitis, papillitis, macular oedema, and retinal haemorrhage. Exclusion criteria were as follows: absence of active posterior uveitis, lack of clear images of the eyes, chorioretinal atrophy, high myopia (spherical equivalent refractive error lower than 8 diopters or axial length ≥28 mm), indication for ocular surgery within 1 year, and presence of other retinal or choroidal diseases.

At every visit, the spherical equivalent refractive error, best-corrected visual acuity, and ocular pressure were measured in all patients. In addition, they underwent a slit-lamp biomicroscopic examination, a fundus examination, and a spectral domain OCT (Spectralis^TM^; Heidelberg Engineering, Heidelberg, Germany). In contrast, FA was performed as necessary, once or twice per year.

The subfoveal choroidal thickness (SCT) was measured with EDI-OCT in the active and the convalescent phase. SCT was defined as the vertical distance at the fovea between the hyperreflective line corresponding to the retinal pigment epithelium and the chorioscleral interface. Digital callipers provided by the Heidelberg Spectralis OCT software version 5.3 were used to measure the SCT.

The extent of FA leakage was graded on a scale of 0 to 3 (0: none, 1: mild, 2: moderate, 3: severe) for the peripheral retina, macula, and optic disc as previously reported^5^. The total FA leakage score was calculated for the active and convalescent phases as the sum of the scores for the peripheral retina, macula, and optic disc. Two experienced uveitis specialists (SS and TK) who were blinded to each other’s assessments and the clinical data of the patients evaluated the FA leakage score independently. If their scores differed, the final score was determined by agreement after discussing the FA images.

In this study, we compared both SCT values and FA leakage scores in the active phase with those in the convalescent phase. Furthermore, we investigated the changes in SCT and FA leakage score between the active and the convalescent phase. The SCT changes were calculated according to the following formula: (SCT in active phase – SCT in convalescent phase)/SCT in active phase × 100 (%). The changes in FA leakage score were defined by the following formula: FA leakage score in the active phase – FA leakage score in the convalescent phase. Additionally, the changes in FA leakage scores at each location of the retina (peripheral retina, macula, and optic disc) and those in SCT were compared between the active phase and the convalescent phase.

For the control group, age-, sex-, and spherical equivalent-matched subjects who underwent EDI-OCT examination because they had advanced cataract with invisible fundus were included from the patients who visited the Department of Ophthalmology at the University of Tokyo Hospital from January 2013 to July 2018. Exclusion criteria for the control group in this study were as follows: (1) abnormal findings in EDI-OCT image, (2) systemic diseases affecting the retina or choroid, and (3) insufficient quality of the EDI-OCT images for measuring the SCT.

Informed consent was obtained from all subjects. This retrospective study was permitted by the Ethics Committee of the University of Tokyo Hospital (No: 2217-(5)) and was conducted in accordance with the tenets of the Declaration of Helsinki.

Clinical characteristics including sex, age, and spherical equivalent refractive error were analysed using the chi-square test, unpaired t-test, and Mann-Whitney U test between groups, respectively. Comparison of the SCT and the FA leakage score between the active and convalescent phases was analysed using the paired t-test and Wilcoxon signed-rank test, respectively. Comparison of the SCT between active phase and control eyes was analysed using the Mann-Whitney U test. The relationship between the changes in SCT and those in FA was evaluated using linear regression analysis. The level of significance was determined as p < 0.05. All statistical analyses were conducted using the Graph Pad Prism Software version 7 (Graph Pad Software, San Diego, California, United States of America).

## Results

A total of 51 eyes in 30 patients were included in the present study after excluding patients from the initial study population of 80 subjects according to the criteria outlined in the methods section. For the control group, 51 eyes of 51 subjects, who were sex-, age-, and spherical equivalent refractive error-matched with those in the BD group, were included (Table 1).

**Table 1:** Clinical characteristics of ocular Bechet’s disease patients and controls.

	BD group	Control group	P value
Number of eyes (Number of subjects)	51 (30)	51 (51)	—
Sex, male: female	20:10	31:20	0.597 *
Age (years), mean ± SD	45.0 ± 13.5	48.1 ± 13.5	0.250 **
Spherical equivalent refractive error (diopters), mean ± SD	−2.66 ± 2.56	−2.46 ± 2.69	0.604 ***
Disease duration (years), mean ± SD	7.35 ± 6.29	NA	—
Interval between active and convalescent phases (months)	7.39 ± 5.17	NA	—

Dashes indicate that no statistical analysis was conducted for the parameters.

* chi-square test, ** unpaired t-test, *** Mann–Whitney U test.

**Figure 1:**
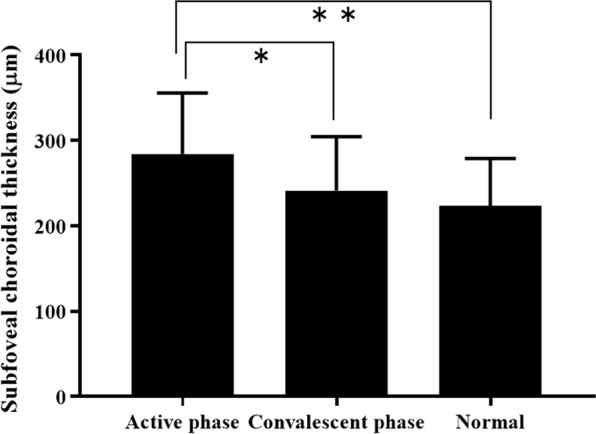
Subfoveal choroidal thickness (SCT) in the active and convalescent phases of Behçet’s uveitis and in normal eyes. SCT is significantly higher in the active phase than in the convalescent phase or in control eyes. Data are presented as mean ± SD. Statistical analyses were performed using the Wilcoxon signed-rank test (SCT in the active phase vs SCT in the convalescent phase, *p < 0.05) and the MannWhitney U test (SCT in the active phase vs SCT in the control eyes, **p < 0.05), respectively.

Figure 1 shows the SCT values determined for the active phase and the convalescent phase in the patient group, and the normal condition in the control group. The mean SCT values were significantly higher in the active phase compared with those in the convalescent phase (284.2 ± 71.5 vs. 241.5 ± 62.9, p < 0.05) and in normal eyes (284.2 ± 71.5 vs. 241.5 ± 62.9, p < 0.05); in contrast, there was no significant difference between BD eyes in the convalescent phase and normal eyes (241.5 ± 62.9 vs. 223.2 ± 55.6, p = 0.170).

**Figure 2:**
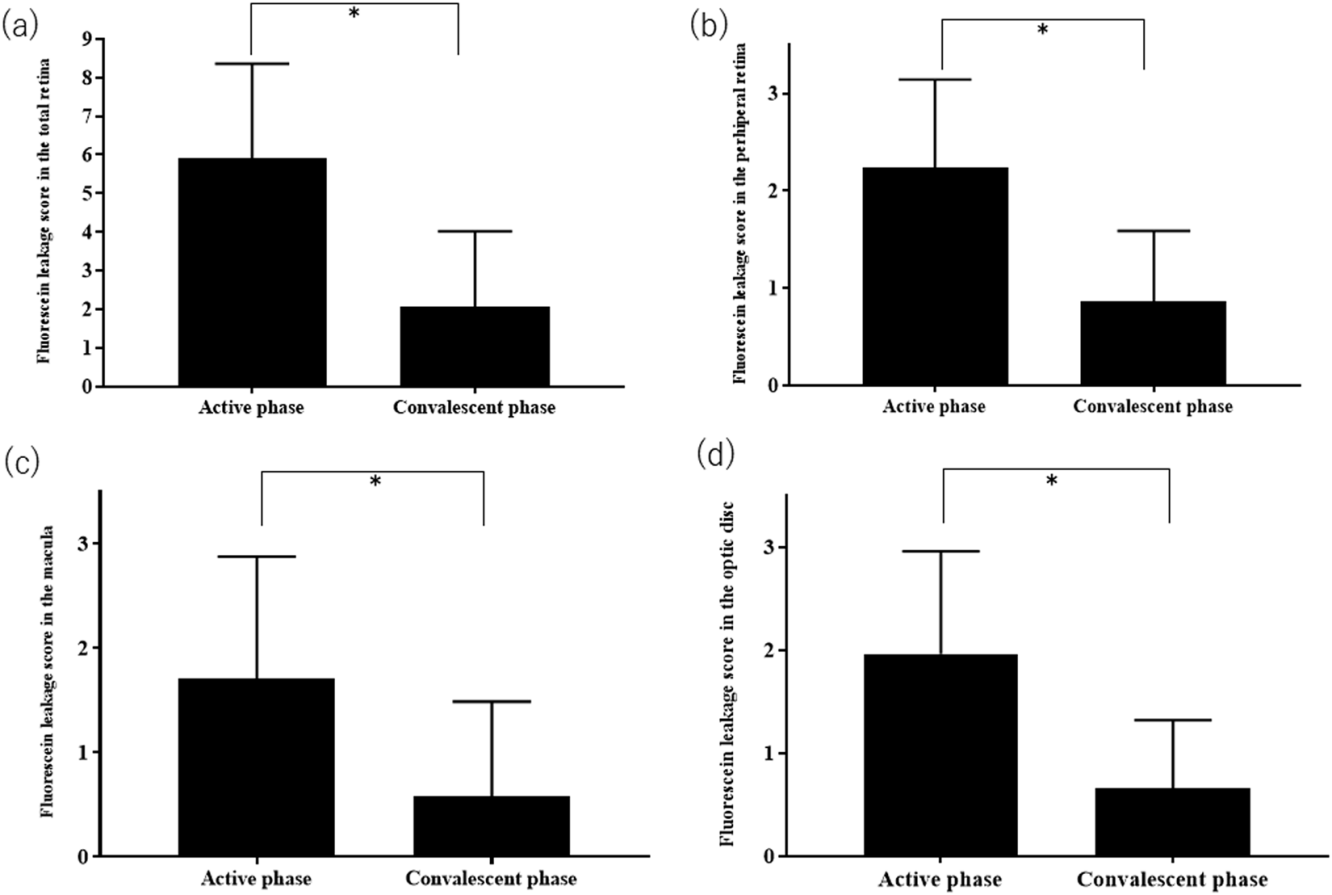
Changes in fluorescein angiography (FA) leakage score in the total retina (**a**), peripheral retina (**b**), macula (**b**), and optic disc (**d**) between the active and convalescent phases. In all areas investigated, the FA leakage scores are significantly higher in the active phase compared with the convalescent phase. Data are presented as mean ± SD. Statistical analyses were performed using the paired t-test (*p < 0.05).

Figure 2 shows the change in FA leakage scores between the active and convalescent phases in the total retina area, peripheral retina, macula, and optic disc. The FA leakage scores in each of these four sites were significantly higher in the active phase than the corresponding scores in the convalescent phase (total retina: 5.90 ± 2.46 vs. 2.06 ± 1.96, p < 0.05; peripheral retina: 2.23 ± 0.91 vs. 0.88 ± 0.73, p < 0.05; macula: 1.71 ± 1.17 vs. 0.89 ± 0.91, p < 0.05; optic disc: 1.96 ± 1.00 vs. 0.67 ± 0.66, p < 0.05).

**Figure 3:**
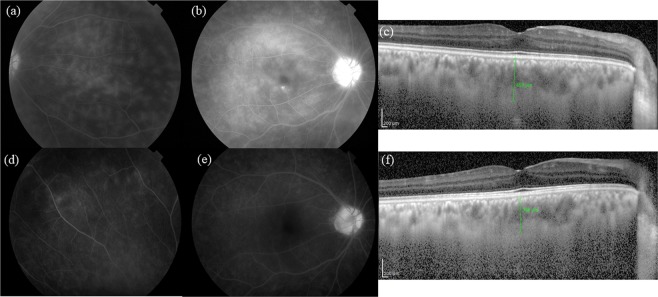
Fluorescein angiography (FA) and enhanced depth imaging optical coherence tomography (EDI-OCT) images of the active and convalescent phases in a representative eye with Behçet’s disease. In the active phase, FA reveals dense retinal vascular leakage in all retinal locations (peripheral retina (**a**), macula (**b**), and optic disc (**b**)), and the subfoveal choroidal thickness (SCT) was determined to be 453 μm (**c**). In the convalescent phase, the retinal vascular leakage improved in all parts (**d**,**e**), while the SCT was decreased at 364 μm (**f**).

Figure 3 shows the FA and EDI-OCT images of the active and convalescent phases in a representative eye of an ocular BD patient. In the active phase, FA demonstrates an extensive dye leakage from retinal vessels and EDI-OCT shows subfoveal choroidal thickening. In contrast, there is only limited dye leakage in FA and reduced SCT in EDI-OCT during the convalescent phase.

**Figure 4:**
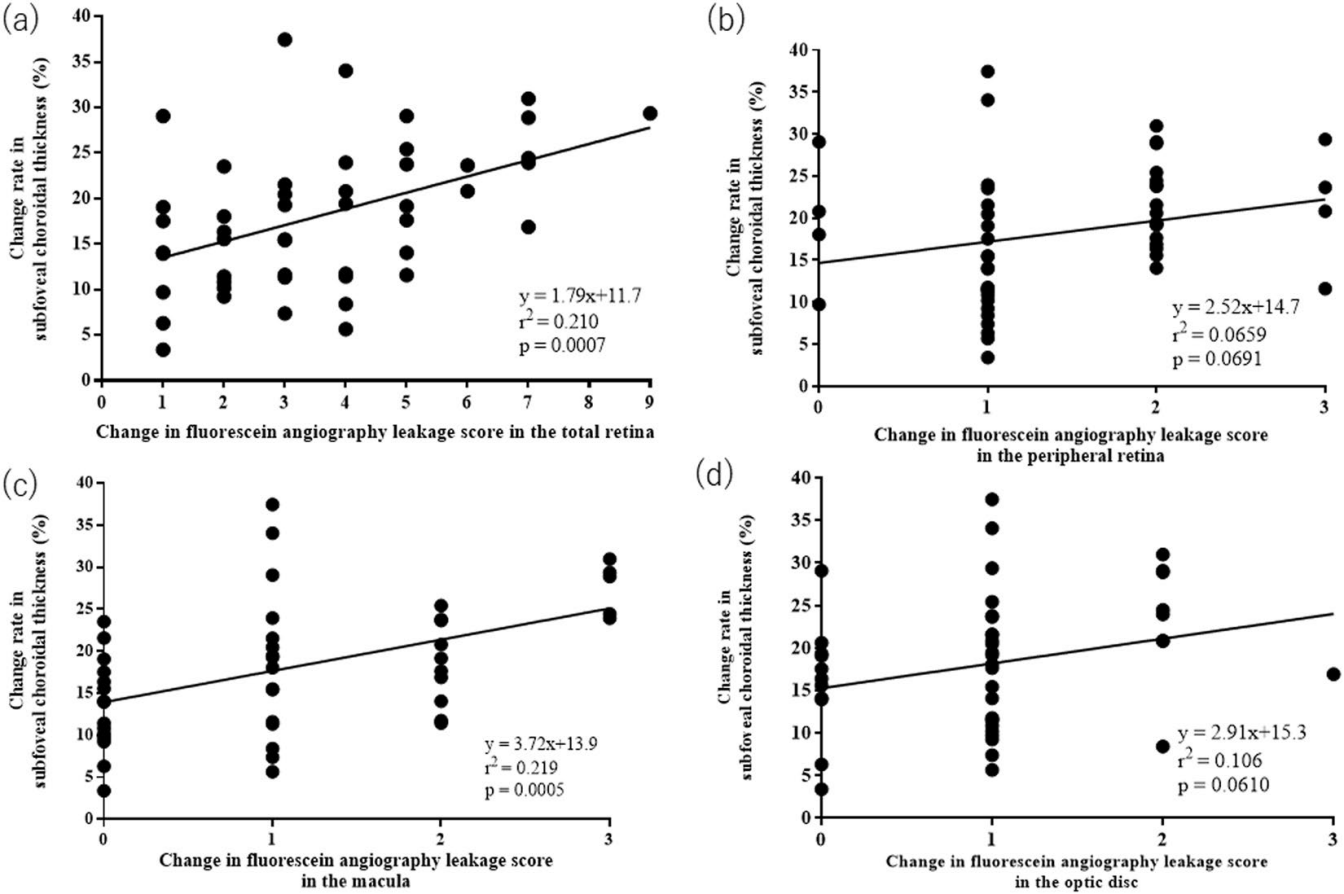
Association between the changes in subfoveal choroidal thickness (SCT) and those in the fluorescein angiography (FA) leakage score of the total retina (**a**), peripheral retina (**b**), macula (**c**), and optic disc (**d**). The associations between these parameters were evaluated using linear regression analysis for the total retina (**a**), peripheral retina (**b**), macula (**c**), and optic disc (**d**). The changes in SCT are significantly correlated with those in the FA leakage score of the total retina (r^2^ = 0.210, p < 0.05, linear regression analysis) and macula (r^2^ = 0.219, p < 0.05).

Next, we examined the relationship between the change rate in SCT and that in FA leakage score (Fig. 4). The SCT change rate (y) correlated significantly with the change (x) in total FA leakage score (y = 1.79X+ 11.7, r^2^ = 0.210, p < 0.05). Finally, we investigated the relationship between the SCT change rate and the changes in FA leakage score according to the location (Fig. 4). In the macula, the SCT change rate was correlated significantly with changes in the FA leakage score (y = 3.72X+ 13.9, r^2^ = 0.219, p < 0.05), while no significant correlations were detected between those parameters for the peripheral retina (y = 2.52X+ 14.7, r^2^ = 0.0659, p = 0.069) or the optic disc (y = 2.91X+ 15.3, r^2^ = 0.106, p = 0.061).

## Discussion

This study investigated the relationship between SCT and FA leakage scores in ocular BD patients. In addition, the FA leakage score was measured in the peripheral retina, macula, and optic disc separately to clarify the locations reflecting the FA leakage and SCT change association. This study clarified that the changes in SCT between the active and convalescent phases are correlated with the corresponding changes in the FA leakage score. This is particularly prominent in the macula.

Previous studies have shown that the choroid is thicker in the acute phase than in the quiescent phase in ocular BD^10,13^ and Vogt-Koyanagi-Harada disease^14,15^ patients. These studies suggest that SCT measurement using EDI-OCT is useful to evaluate and monitor the disease activities in uveitis. In contrast, a previous retrospective study reported that infliximab therapy reduces vascular leakage as established using FA^5^. Vascular FA leakage has also been useful to evaluate the disease activity in ocular BD patients^5^.

As expected, the current study demonstrated that both SCT and FA leakage scores in ocular BD patients were significantly higher in the active phase compared with the convalescent phase (Fig. 1). For FA leakage, this finding was confirmed for all investigated retinal locations, i.e., the peripheral retina, macula, and optic disc (Fig. 2). Our investigation of the relationship between the changes in SCT and FA leakage scores in the active and convalescent phases demonstrated that the changes in vascular FA leakage from the macula, but not the peripheral retina or the optic disc, were significantly correlated with the corresponding changes in SCT (Fig. 4).

It is well-known that atrophy of the macula or optic disc is one of the complications leading to vision deterioration in ocular BD patients^16^. Macular and optic disc atrophy are assumed to be attributed to retinal vasculitis and papillitis, which can be identified using FA^17^. The current study demonstrates that SCT measurement may be a useful tool to monitor the inflammatory activities in the posterior pole of the fundus, raising the possibility to detect early signs of macular atrophy in ocular BD patients. Furthermore, SCT is a non-invasive examination technique that does not depend on the use of a fluorescein dye. Consequently, SCT measurement using EDI-OCT may be a valuable choice to evaluate retinal vasculitis near the macula in BD uveitis.

This study has several limitations. First, this investigation was conducted retrospectively. Second, the study results may not be applicable to subjects who have undergone ocular surgery or those with chorioretinal atrophy, highly myopic eyes, and other retinal or choroidal diseases. Third, this study was performed at a single institute, and the number of enrolled study subjects was low. Further investigations are necessary to clarify the site-specific relationship of SCT and leakage in FA in BD uveitis.

In conclusion, SCT and FA leakage scores were significantly increased in the active compared with the convalescent phase. The changes in SCT were significantly associated with the changes in FA leakage scores, especially in the macula region. Therefore, SCT measurement may be a potentially valuable and non-invasive approach for evaluating inflammation near the macula in Behçet’s uveitis.

## Data Availability

The datasets used and analysed during the current study can be obtained from the corresponding author on reasonable request.
